# Children Allergic to Peanut and/or Tree Nuts Are at Risk of Accidental Allergic Reactions

**DOI:** 10.1111/cea.70132

**Published:** 2025-09-01

**Authors:** Marina Vasse, Marie Moyart, Camille Cisterne, Caroline Thumerelle, Elodie Drumez, Hélène Béhal, Guillaume Pouessel, Stéphanie Lejeune, Antoine Deschildre

**Affiliations:** ^1^ Pediatric Pulmonology and Allergy Department CHU Lille Lille France; ^2^ Department of Biostatistics CHU Lille Lille France; ^3^ Department of Paediatrics Children's Hospital Roubaix France; ^4^ Univ Lille, ULR 2694: METRICS Lille France; ^5^ Univ. Lille, CNRS, Inserm, CHU Lille, Institut Pasteur de Lille, U1019—UMR 9017—CIIL—Center for Infection and Immunity of Lille, Pediatric Pulmonology and Allergy Department Hôpital Jeanne de Flandre Lille France

**Summary**

- This study highlights the risk of AAR in peanut and TN allergic children.
- It confirms the limits of a management based exclusively on avoidance.

To the Editor,

Despite improvements in food allergy (FA) management, the risk of accidental allergic reaction (AAR) including life‐threatening anaphylaxis and fatalities, is remaining with consequences on quality of life [1, 2]. It has been estimated as roughly 10% to 12% per year for peanut allergy [3, 4]. In France, the risk of recurrent accidental allergic reaction to food was confirmed in 39 children with a history of FA admission to a paediatric intensive care unit for anaphylaxis. In all, 70% of these children experienced at least one recurrent reaction during a follow‐up period from 4 to 14.1 years [5]. Another prospective study followed 196 children with various FA (peanut *N* = 62%; TN *N* = 60%) over a year and found that 31.1% experienced at least one accidental ingestion (peanut *N* = 9; TN *N* = 7), with some reporting up to nine. 31.6% of reactions led to anaphylaxis—highlighting the value of regular monitoring and education [6]. However, real‐life data in peanut and/or TN allergic children on accidental reactions and risk factors remain scarce. Better knowledge is needed to improve management, including prevention and new treatment strategies. The aim of this study was to determine the incidence of AAR related to peanut and tree nut (TN) ingestion in children (aged 0–18 years) with a physician‐diagnosed peanut and/or TN allergy, followed at the Lille University paediatric allergy tertiary care centre (France), and to describe the characteristics of these AAR. This prospective study with a 1‐year follow‐up was approved by an ethics committee (SUD EST VI, reference: 2021‐A00746‐35) and patients provided consent.

The patients were enrolled in the study during a scheduled follow‐up visit. Patients' characteristics were recorded, including where appropriate data on treatment with peanut oral immunotherapy (OIT) in patients with peanut allergy. The objective of the study was explained in detail to parents when their children were enrolled in order to obtain all data in the event of an AAR. The families were then contacted every 3 months by email and/or phone. If an accidental reaction had occurred, data were collected using a structured questionnaire: severity of the allergic reaction according to the World Allergy Organisation (WAO) 2020 grading system, culprit allergen, details on food products, food amount, location of the AAR, cofactors, and management.

Of the 191 children initially enrolled in our study, 44 were excluded because of no or incomplete follow‐up. In the 147 included patients, the diagnosis of PN and/or TN allergy was based on a history of accidental reaction in 105 or by positive OFC in the remaining 42. In all, 81 had peanut allergy (55%), 34 TN allergy (23%), and 32 both allergies (22%). Forty‐eight peanut allergic patients (42%) were treated with peanut OIT. Twenty‐six reactions were recorded in 25 patients. Two AAR triggered two times by hazelnut ingestion occurred in one child. Four reactions were triggered by contact without ingestion and two reactions by other foods than peanut and TN. Three reactions were directly related to peanut OIT dose. Finally, 17 AAR (anaphylaxis in 5 cases) following accidental ingestion in 16 children were analysed, triggered by peanut in 8 cases (7 patients allergic to peanut only and one patient allergic to both peanut and TN) and by TN in nine cases (hazelnut, 7; pistachio, 2). Six patients (38%) were younger than 6 years, six (38%) were aged between 6 and 12 years, and four (25%) were older than 12 years. There were no significant differences between the characteristics of the patients with and without any accidental reactions (data not shown). The characteristics of the 17 reactions are presented in Table 1. The annual incidence of reactions triggered by accidental ingestion of either peanut or TN was 10.9% (95% CI: 6.4% to 17.1%); this rate was 8.6% (95% CI: 3.6% to 17%) in peanut allergic patients, 8.8% (95% CI: 1.8% to 23.7%) in TN allergic patients, and 18.7% (95% CI: 7.2% to 36.4%) in patients allergic to both peanut and TN.

**TABLE 1:** Characteristics of the study population (part A) and of the real‐life AAR (part B).

	All patients *n* = 147	Peanut allergy *n* = 81	TN allergy *n* = 34	Peanut + TN allergy *n* = 32
(A) *Characteristics of the population at inclusion*				
Gender *N* (%)				
Male	86 (59)	48 (60)	20 (59)	18 (56)
Median age, years (range)	8 (2–17)	8 (1–18)	7 (1–16)	9 (2–17)
Allergic comorbidities *N* (%)				
Atopic dermatitis	98 (67)	50 (60)	24 (71)	23 (79)
Asthma	97 (66)	51 (60)	26 (76)	19 (66)
Rhinitis	47 (32)	29 (35)	11 (32)	7 (78)
Other food allergy	30 (20)	11 (14)	7 (21)	12 (38)
History of real‐life allergic reaction N (%)	105 (71)	61 (75)	22 (65)	22 (69)
Median age at the most serious accidental reaction, years (range)	3 (1–11)	2 (1.5–13)	3.1 (0.6–11)	3 (1–11)
(B) *Characteristics of the 17 real‐life accidental reactions*				
Patient with 1 reaction	15	6	3	6
Patients with 2 reactions	1	0	0	1
Patients with peanut OIT	1	1	0	0
Gender (%)				
Male	8 (50)	3 (43)	2 (67)	3 (50)
Median age at reaction, years (range)	7 (2–15)	7 (2–13)	7 (4–7)	7 (3–15)
Food trigger *N* (%)				
Peanut	8 (47)	7 (100)	0	1 (14)
Pistachio	2 (12)	0	1 (33)	1 (14)
Hazelnut	7 (41)	0	2 (67)	5 (72)
Severity (WAO) *N* (%)				
1	7 (41)	3 (43)	1 (33)	3 (43)
2	5 (29)	0	2 (66)	3 (43)
3	4 (24)	4 (57)	0	0
4	1 (6)	0	0	1 (14)
5	0	0	0	0
Location *N* (%)				
Home	8 (47)	2 (29)	1 (33)	5 (72)
School	1 (6)	1 (14)	0	0
Childcare	2 (12)	1 (14)	1 (33)	0
Restaurant	1 (6)	1 (14)	0	0
Friend/family	2 (12)	1 (14)	0	1 (14)
Other	3 (18)	1 (14)	1 (33)	1 (14)
Treatment *N* (%)				
Antihistamines only	11 (65)	3 (43)	3 (100)	5 (72)
Inhaled short‐acting β2 agonists	2 (12)	1 (14)	0	1 (14)
Adrenaline autoinjector	2 (12)	0 (10)	0	2 (29)
Health‐care use *N* (%)				
Emergency department	2 (12)	1 (14)	0	1 (14)
General practitioner	2 (12)	2 (29)	1 (33)	0
Hospitalisation	0	0	0	0

*Note:* The food products involved in the AAR were identified in 16 cases: sweets (two peanut, five hazelnut‐related cases), cereal bars (two peanut‐related cases), hazelnut spread (two hazelnut related‐cases), oriental pastry (one pistachio‐related case). The last cases were related to crude or crushed peanut (two peanut related cases) or pistachio (one pistachio related case).

We have shown that despite a follow‐up in a referral centre and education, AAR to peanut or TN, including anaphylaxis (29%), occurs in children of all ages, including the youngest, and frequently outside home (47%). They could be related to food products with declared peanut or TN on the ingredient list, indicating a lack of vigilance. Excluded reactions related to peanut OIT doses, the incidence of AAR related to peanut (8.8%) and to TN (8.6%) ingestion were very close and higher if there was an association of both allergies (18.7%). AAR to TN were mainly observed with hazelnut, one of the main TN allergies in France [7]. We also observed that adrenaline was not used enough, but we were unable to identify any risk factors, particularly in terms of age or history of previous AAR (data not shown). Although that was not the aim of our work, we have observed that children treated with peanut OIT had treatment‐related reactions, especially during the up‐dosing. This confirms the known, ‘controlled’ risk of reaction associated with OIT, decreasing over time [8]. On the other hand, peanut OIT limits the risk of real‐life AAR [9], recorded in only one of the 48 patients on OIT in our cohort. Finally, we are aware of the limitations of this study, which was conducted in a referral centre with incomplete data due to loss to follow‐up or incomplete follow‐up in 23% of enrolled patients. Furthermore, 1 year may not be a long enough period to observe accidental and unpredictable reactions. However, we felt that this duration was sufficient to cover a representative period of the child's life, both within and outside the family.

Our study confirms the limits of the management based exclusively on avoidance. This highlights not only the need for more education on diet and emergency management but also for new strategies to limit this risk of AAR [9].

## Author Contributions

M.V., A.D.: conceptualisation, data extraction, analysis, writing, review and approval of the final manuscript. M.M., C.C., C.T., S.L., G.P.: review and approval of the final manuscript. E.D., H.B.: conceptualisation, statistical analysis, writing, review and approval of the final manuscript.

## Conflicts of Interest

Guillaume POUESSEL: Bausch & Lomb, Meda/Mylan/Viatris, Stallergenes Greer, Novartis, ALK‐Abello, DVB Therapeutics, AImmune Therapeutics/Nestlé, Theravia/CTRS/Admedica: scientific work or speaker/consulting fees. Antoine DESCHILDRE: Sanofi Aimmune Therapeutics, DBV Technologies, ALK, Stallergenes Greer, AstraZeneca, GSK, Novartis, Sanofi, Regeneron Pharmaceuticals Inc., Viatris, Celltrion–speaker/consulting fees outside the submitted work.

## Data Availability

Data sharing not applicable to this article as no datasets were generated or analysed during the current study.
